# The brain targeted delivery of programmed cell death 4 specific siRNA protects mice from CRS-induced depressive behavior

**DOI:** 10.1038/s41419-021-04361-9

**Published:** 2021-11-12

**Authors:** Yufeng Jia, Xiao Zhuang, Yi Zhang, Ming Zhao, Nuo Chen, Wen Li, Faliang Zhu, Chun Guo, Yan Li, Qun Wang, Yuan Li, Lining Zhang

**Affiliations:** 1 Shandong Key Laboratory of Infection and Immunity, Department of Immunology, School of Basic Medical Sciences, Shandong University, 250012 Jinan, Shandong China; 2 Department of Pathogenic Biology, School of Basic Medical Science, Shandong University, 250012 Jinan, Shandong China

**Keywords:** Neuroimmunology, Depression

## Abstract

Depression is one of the most common psychiatric disorders. Recently, studies demonstrate that antidepressants generating BDNF not only maintain synaptic signal transmission but also repress neuroinflammatory cytokines such as IL-6 and IL-1β. Therefore, promoting BDNF expression provides a strategy for the treatment of depression. Our recent research has indicated that programmed cell death 4 (Pdcd4) is a new target for antidepressant treatment by facilitating BDNF. Herein, we modified Pdcd4 specific small interfering RNA (siPdcd4) with the rabies virus glycoprotein peptide (RVG/siPdcd4) which enables it cross the blood-brain barrier (BBB). We found that RVG/siPdcd4 complex was selectively delivered to neurons and microglia and silenced the expression of Pdcd4, thereby up-regulating the level of BDNF and down-regulating IL-6 and IL-1β expression. More importantly, RVG/siPdcd4 injection attenuated synaptic plasticity impairment and protected mice from CRS-induced depressive behavior. These findings suggest that RVG/siPdcd4 complex is a potential therapeutic medicine for depression.

## Introduction

Major depressive disorder (MDD) is one of the most common psychiatric disorders and causes a leading disease burden [1]. The limitation of monoamine theory in practical treatment makes people realize that neurotrophin is indispensable to depression treatment, and accumulating evidence has supported the critical role of brain-derived neurotrophic factors (BDNF) in emotion regulation [2]. Recently, considerable efforts have been made in regulating the expression of BDNF for therapeutic applications in depression [3]. There is also evidence that the dysfunction or mutation of BDNF gene leads to susceptibility to environment stress, while BDNF recombinant protein injection corrects mice depressive behaviors which are caused by chronic stress [4–7]. Moreover, prevalence antidepressants such as selective serotonin reuptake inhibitors (SSRIs), selective noradrenalin reuptake inhibitors (SNRIs), ketamine, and scopolamine alleviate depression by enhancing BDNF production [8–10]. Therefore, enhancing the expression of BDNF is an important strategy for depression therapy. In addition, the increasing evidence indicates that stress-induced pro-inflammatory cytokines secretion is also involved in the development of depression. The pro-inflammatory cytokines such as interleukin-6 (IL-6), interleukin-1β (IL-1β), and tumor necrosis factor-α (TNF-α) in peripheral and central nervous system are significantly increased in patients with depression [11]. In summary, these studies suggest that the combination of promoting BDNF expression in depressive patients and inhibiting pro-inflammation plays a critical role in antidepressant development. Thus, designing a method to target the molecule which plays a key role in BDNF expression and neuroinflammation is highly desirable.

Programmed cell death 4 (Pdcd4) as a tumor suppressor has been discovered for several years. Pdcd4 inhibits translation initiation of protein indirectly by competitively combining with Eukaryotic Initiation Factor 4A (eIF4A) [12]. The co-crystal structures of eIF4A and the MA3 domains of Pdcd4 has revealed that Pdcd4 inhibits several gene translation, such as P53, c-Myb [13, 14]. Due to the translational repressor function of Pdcd4, we found its negative role in depression. Especially, knockout of Pdcd4 directly upregulates BDNF expression at translational level and reverses CRS-induced depression-like behaviors [15]. Therefore, Pdcd4 is a new target for depression treatment, and silencing the expression of Pdcd4 in central nervous system provides a theoretical basis for the antidepressant drugs discovery.

Small interfering RNA (siRNA)-mediated gene knockdown offers a therapeutic strategy to overcome neurological disorder. For the benefit of small molecular weight, flexible application and less immune response, siRNA has been applied to neuropathy treatment [16, 17]. In 2018, FDA approved the first RNA interference drug, Patisiran, which selectively silences transthyretin (TTR) expression and has been applied to familial amyloid polyneuropathy (FAP) treatment. However, limited access of targeting gene of interest into the brain needs to be solved by developing siRNA delivery system. The infection specificity of rabies virus mainly depends on its surface rabies virus glycoprotein (RVG), which specifically binds to the acetylcholine receptor (AchR) on nerve cells [18]. RVG-9dR peptide has emerged as a promising ligand for the drug delivery into central nerve system [19]. With the help of RVG-9dR peptide, siRNA is effectively delivered into neuronal cells which expressing acetylcholine receptors (AchR) through circulation system [20, 21]. Increasing researches have shown that RVG-9dR peptide-coupled siRNA system has been used for the treatment of neurological disorders. For example, the administration of RVG-bounded tumor necrosis factor specific siRNA (siTNF-α) alleviates neuroinflammatory disorder which caused by bacteria infection [22]. In addition, the RVG/siRNA system mediated clearance of BACE1 and α-synuclein significantly reverses neurodegeneration diseases, and those have been utilized into clinical trial [23, 24]. Though brain-targeting siRNA system has been feasible, lacking specific gene target narrows gene therapy for depression.

In this study, we explicitly addressed the gene-targeting strategy for depression treatment. Based on our previous research, we found hippocampal local injection of lentivirus-carried siPdcd4 could rescue CRS-induced depression on mice [15]. To make siPdcd4 clinically feasible, we did the following experiments. First, we characterized the specificity and validity of Pdcd4 siRNA in vitro, and found that siPdcd4 promoted BDNF expression and inhibited the expression of IL-6 and IL-1β in brain-derived cells. To make siRNA brain accessible, the siPdcd4 was modified with RVG-9dR peptide, and was proved that it was able to be selectively delivered to nerve cells. Furthermore, we traced the dynamic delivery process of RVG/siPdcd4 from peripheral to brain in mice, and tested the down-regulation of Pdcd4 in brain. Finally, we found that the treatment of RVG-modified siPdcd4 showed obviously anti-depressive behavior by inhibiting inflammatory response, up-regulating BDNF expression and correcting neuronal plasticity. That finding identifies a potentially powerful and clinically feasible approach to depression therapeutic.

## Materials and methods

### Animals

Wild-type C57BL/6J male mice aged 4–6 weeks (weight 22–25 g) were used in the animal experiments. All mice were purchased from Vital River Laboratory Animal Technology Co. Ltd (Beijing, China) and carefully reared in SPF laboratory of animal experimental center of Shandong University.

### Chronic restraint stress

Mice were put into ventilated polypropylene bound stress tube during 9:00–11:00 a.m. every day, and the mice in control group were grabbed at same time. The food and water were deprived during experiment.

### Tail suspension test

Depressive-like phenotypes in these models were validated by tail suspension test (TST), and the detail protocol is related to Supplementary materials and methods.

### Forced swimming test

Depressive-like phenotypes in these models were validated by forced swimming test (FST), and the detail protocol is related to Supplementary materials and methods.

### Sucrose preference test

Depressive-like phenotypes in these models were validated by sucrose preference test, and the detail protocol is related to Supplementary materials and methods.

### Peptide and siRNA modification

For the preparation of siPdcd4 with RVG-9dR peptide complex (RVG/siPdcd4), the mixture was generated following the protocol which is related to Supplementary materials and methods.

### In vivo imaging assay

The detail steps are related to Supplementary materials and methods.

### Animal injection of RVG/siRNA complex

Applying the RVG/siRNA complex for animal, the detail steps of experiment was illustrated in Supplementary materials and methods.

### Cell culture and siRNA transfection

SH-SY5Y,BV2, HT-22, Hela cells, and the primary hippocampal neurons were used in this report, and the detail cell culture protocol is related to Supplementary materials and methods.

### Western blot

The cell and brain tissue lysates were used for western blot assay, and the detail protocol is related to Supplementary materials and methods.

### Real-time PCR

Relative target gene mRNA expression was normalized to β-actin mRNA and calculated using the $\Delta$Ct method. The detail protocol and sequences of primers in experiment were related to Supplementary materials and methods.

### ELISA

The concentration of IL-6, IL-1β, and BDNF were quantified by specific Enzyme-Linked Immunosorbent Assay (ELISA) kits (Biolegend, CA, USA) according to the manufacturer’s instructions. The detail steps are related to Supplementary materials and methods.

### High-content imaging analysis

The cells were fixed in paraformaldehyde solution for 15 min, and cells nuclear were stained with DAPI for 10 min at room temperature. All the images were captured with Laser confocal high content imaging analysis system (PerkinElmer, MA, USA).

### Golgi staining

The changes of synaptic plasticity in mice were evaluated by golgi staining, and the detail steps are related to Supplementary materials and methods.

### Statistical analysis

The statistical analysis was performed using PrismGraphPad 7 Software. The analysis method is related to Supplementary materials and methods.

## Results

### Pdcd4 siRNA upregulates BDNF expression and suppresses inflammatory response in cells of nerve system

**Fig. 1:**
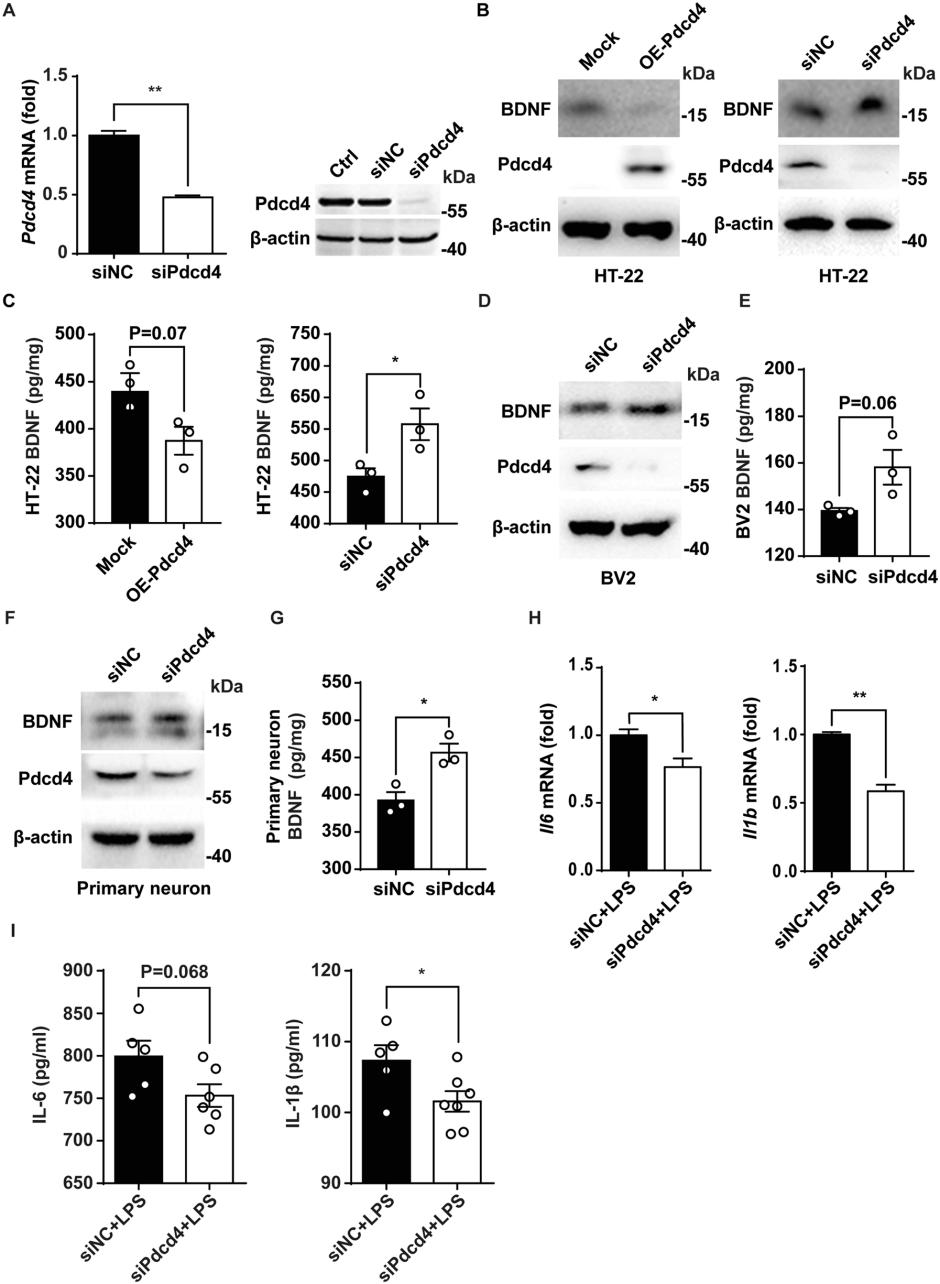
Pdcd4 siRNA upregulates BDNF expression and suppresses inflammatory response in cells of nerve system. **A** The interference efficiency of Pdcd4 small interfering RNA (siPdcd4) were detected by RT-PCR (*n* = 3 per group) and western blot. **B**, **C** Pdcd4-FLAG vector (2 μg) and Pdcd4 specific siRNA (100 pM) were transfected into mouse hippocampal cell line HT-22, the expression changes of BDNF in cell lysis were measured by western blot and ELISA (*n* = 3 per group). **D**–**G** Pdcd4 specific siRNA was transfected into mouse microglia cell line BV2 and primary hippocampal neurons by lipofectamine 2000 (100 pM siRNA per well in six-well plate). The interference efficiency of Pdcd4 was detected by western blot and the expression changes of BDNF in cell lysis were measured by western blot and ELISA (*n* = 3 per group). **H** Pdcd4 specific siRNA (100 pM) was transfected into mouse microglia cell line BV2 by lipofectamine2000. After 24 h, lipopolysaccharide (10 ng/ml for 8 h) and ATP (2.5 mM for 40 min) were added to induce microglia to secrete pro-inflammatory cytokines, and the mRNA changes of pro-inflammatory cytokines IL-6 and IL-1β (*n* = 3 per group) were measured by RT-PCR. **I** The concentration of IL-6 (siNC+LPS *n* = 5, siPdcd4+LPS *n* = 6) and IL-1β (siNC+LPS *n* = 5, siPdcd4+LPS *n* = 7) in culture supernatant were measured by ELISA. Values are indicated as mean ± SD, Student’s *t* test *p < 0.05, **p < 0.01. All the experiments were repeated at least three times independently.

Our recent research found that Pdcd4 is an endogenous suppressor of BDNF, meaning that silencing Pdcd4 will increase the expression of BDNF in nerve cells [15]. At first, we prepared the Pdcd4 specific siRNA (siPdcd4) and the interference efficiency of Pdcd4 siRNA was tested. The results showed that Pdcd4 siRNA effectively silenced Pdcd4 expression at mRNA and protein levels (Fig. 1a). Overexpression of Pdcd4 in mouse hippocampal neuron cell line HT-22 significantly decreased the expression of BDNF, while knocking-down of Pdcd4 by siPdcd4 increased the expression of BDNF which detected by western blot and ELISA (Fig. 1b, c). Moreover, siPdcd4 also promoted BDNF expression in BV2 and primary hippocampal neurons (Fig. 1d–g). According to the inflammation hypothesis of depression, microglia-mediated inflammatory response accelerates nerve damage in depression [25]. We tested the effect of Pdcd4 knockdown on the expression of inflammatory cytokines in microglia. The results showed that siPdcd4 significantly suppresses the secretion of pro-inflammatory cytokines IL-6 and IL-1β under lipopolysaccharide (LPS) stimulation (Fig. 1h, i). These results suggest that siPdcd4 can up-regulate the expression of BDNF in nerve cells and inhibit the secretion of pro-inflammatory cytokines in microglia.

### RVG-9dR peptide allows delivery siPdcd4 into neuron and microglia

**Fig. 2:**
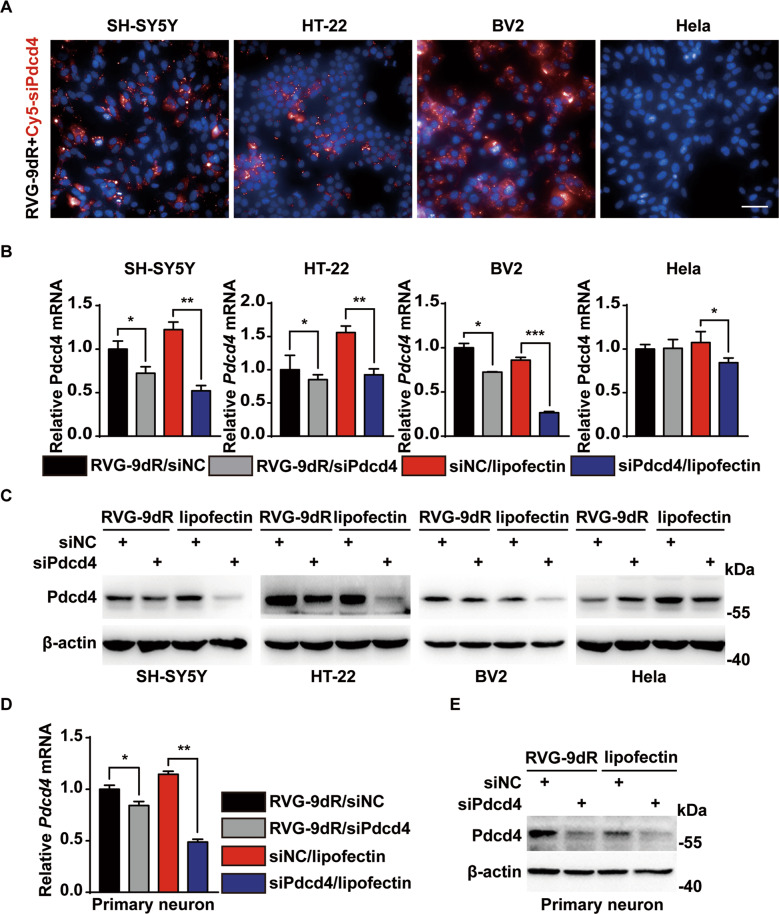
RVG-9dR peptide allows delivery siPdcd4 into neuron and microglia. **A** The Cy5 fluorescent-labeled RVG/siPdcd4 (RVG/Cy5-siPdcd4) was added to infect SH-SY5Y, HT-22, BV2 and Hela cells for 12 h, and the red fluorescence distribution in cells were detected by high-content imaging system. As pictures shown DAPI staining (Blue) and Cy5-siPdcd4 (red). Scale bar, 50 μm. **B**–**E** The cells (Hela, SH-SY5Y, HT-22, BV2, and primary neuron) were treated with RVG/siNC, RVG/siPdcd4 or lipofectamine/siRNA (100 pM siRNA per well) for 24 h, and the interference efficiency of siPdcd4 were measured by RT-PCR (*n* = 3 per group) and western blot. Values are indicated as mean ± SD, Student’s *t* test *p < 0.05, **p < 0.01, ***p < 0.001. All the experiments were repeated at least three times independently.

Our previous results indicate that silencing of Pdcd4 expression in hippocampus has antidepressant effect in depressive mice, suggesting siPdcd4 can be applied for depression therapy as an antidepressant. How to cross the BBB is an important obstacle of siRNA drug delivery for mental illness therapy. Previous studies have found that siRNA mixture with 9dR peptide-modified rabies virus-derived-RVG (RVG-9dR) can realize nerve cells targeting [21]. To test whether siPdcd4 effectively crosses the BBB and enters into nerve cells, Cy5 (red) was labeled on siPdcd4. RVG/Cy5-siPdcd4 were detected in different cells by imaging system. The results showed that RVG/Cy5-siPdcd4 could selectively enter to nerve cell lines which express acetylcholine receptor (AchR) including SH-SY5Y, HT-22, and BV2 but not to non-neural cells such as cervical cancer cell line Hela (Fig. 2a). To tested the interference efficiency of RVG/siPdcd4, the changes of Pdcd4 expression were detected by RT-PCR and western blot, and the lipofectamine-carried siPdcd4 intervention group was set as positive control. As shown as in Fig. 2b, c, we found lipofectamine-carried siPdcd4 silenced the mRNA and protein expression of Pdcd4 in all detected cell line including Hela cell. However, RVG/siPdcd4 only knockdown the expression of Pdcd4 in nerve cells (SH-SY5Y, HT-22, and BV2). Moreover, we found that Pdcd4 was effectively silenced in primary hippocampal neurons after RVG/siPdcd4 intervention (Fig. 2d, e). Overall, the results suggest that siPdcd4 specifically enter brain-derived cells and successfully knockdown the expression of Pdcd4 with the help of RVG-9dR peptide.

### RVG/siPdcd4 facilitates BDNF expression and suppresses inflammatory response

**Fig. 3:**
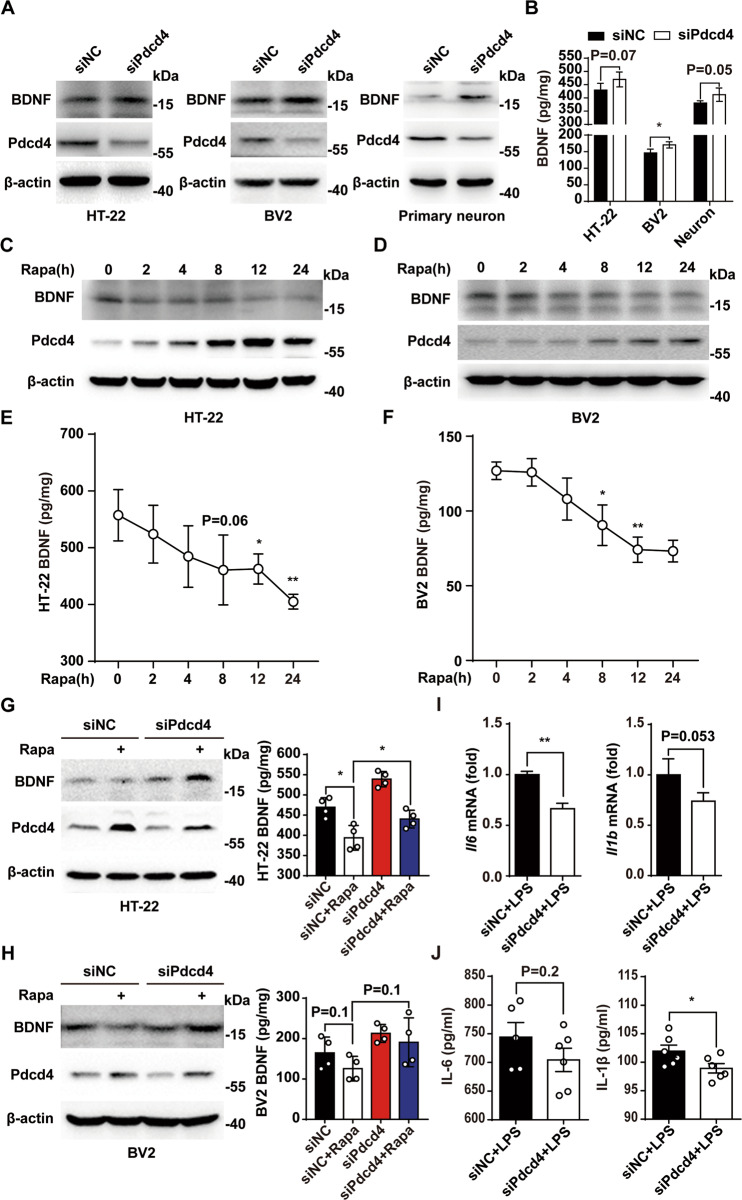
RVG/siPdcd4 facilitates BDNF expression and suppresses inflammatory response. **A**, **B** RVG/siNC and RVG/siPdcd4 (100 pM) were used to infect HT-22, BV2, and primary hippocampal neuron. After 24 h, the Pdcd4 and BDNF expression changes in cell lysis were measured by western blot and ELISA. **C**–**F** The mTORC1 specific inhibitor rapamycin (200 mM) were used to blocking mTORC1 activation in HT-22 and BV2 cells, and the changes of BDNF and Pdcd4 expression in different time points of cell were measured by western blot and ELISA (*n* = 4 per group). **G**, **H** RVG/siPdcd4 (100 pM) were used to slience HT-22 and BV2 Pdcd4 expression. After 24 h, rapamycin (200 mM for 8 h) was added to inhibit mTORC1 activation, and the expression changes of Pdcd4 and BDNF in cell were measured by western blot and ELISA (*n* = 4 per group). **I** RVG/siPdcd4(100 pM) was used to slience BV2 cells Pdcd4 expression. After 24 h, lipopolysaccharide (10 ng/ml for 8 h) and ATP (2.5 mM for 40 min) were used to stimulate the up-regulation of pro-inflammatory cytokines, and the mRNA changes of IL-6 and IL-1β were measured by RT-PCR (*n* = 3 per group). **J** The concentration of IL-6 (siNC+LPS *n* = 5, siPdcd4+LPS *n* = 6) and IL-1β (*n* = 6 per group) in supernatant were measured by ELISA. Values are indicated as mean ± SD, Student’s *t* test *p < 0.05, **p < 0.01. All the experiments were repeated at least three times independently.

In order to prove the facilitation of RVG/siPdcd4 on BDNF expression. We detected the changes of BDNF in brain-derived cells (HT-22, BV2 and primary neuron) by western blot and ELISA, and the results showed that BDNF were significantly up-regulated in RVG/siPdcd4 group compared with RVG/siNC group (Fig. 3a, b). Moreover, the increase of mTORC1 activation caused by the rapid-antidepressant Ketamine promotes the expression of BDNF [26, 27]. To mimic the effect of mTORC1 activity changes in depression in vivo, HT-22 and BV2 were treated with mTORC1 inhibitor rapamycin (200 nM), and then the changes of Pdcd4 and BDNF expression were measured by western blot and ELISA. In consistent with the results in vivo, the expression of BDNF was decreased with the time course of rapamycin administration, following by the up-regulated expression of Pdcd4 (Fig. 3c–f). To explore whether RVG/siPdcd4 induces BDNF expression at mTORC1 inhibitory condition, RVG/siPdcd4 was added to nerve cells to silence endogenous Pdcd4 for 24 h, and then rapamycin (200 nM) was administrated to block mTORC1 activation for 8 h. The results showed that RVG/siPdcd4 significantly reversed the inhibition of rapamycin on BDNF expression in HT-22 and BV2 cells (Fig. 3g, h). Next, we tested the effect of RVG/siPdcd4 on the expression of inflammatory cytokines in BV2. We found that RVG/siPdcd4 inhibited the expression of IL-6 and IL-1β in BV2 stimulated by lipopolysaccharide (LPS) (Fig. 3i, j). These results suggest that RVG/siPdcd4 promotes BDNF expression and inhibits pro-inflammatory cytokines secretion in neuron and microglia.

### RVG/siPdcd4 is able to enter brain by intravenous injection and successfully stimulates BDNF expression

**Fig. 4:**
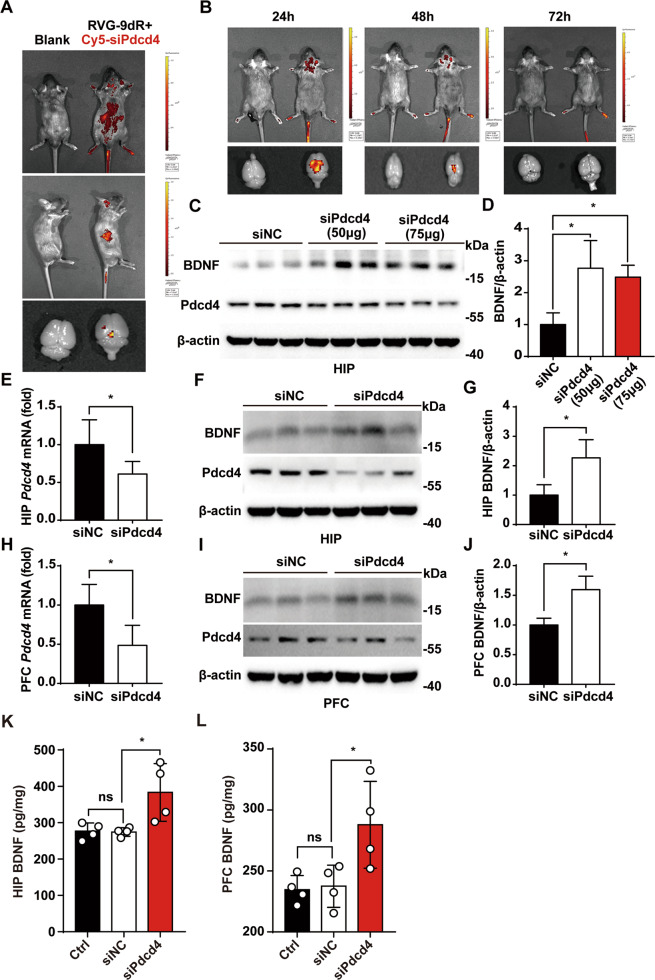
RVG/siPdcd4 is able to enter brain by intravenous injection and successfully stimulates BDNF expression. **A** RVG/Cy5-siPdcd4 (50 μg) was dissolved in 200 μl 5% glucose solution and injected into mice through the tail vein. After 6 h, the distribution of red fluorescence was detected by small animal three-dimensional live imaging system. **B** RVG/Cy5-siPdcd4 (50 μg) was injected into mice via tail vein, the intensity and distribution of red fluorescence were detected by the small animal three-dimensional live imaging system at 24 h, 48 h, and 72 h after injection. **C**, **D** RVG-9dR modified siNC (50 μg,2 mg/kg), siPdcd4 (50 μg,2 mg/kg), and siPdcd4 (75 μg,3 mg/kg) were injected into mice (*n* = 3 per group) through the tail vein. Fresh hippocampal tissue was extracted 48 h later, the Pdcd4 interference effect and BDNF expression changes were detected by western blot. **E**–**J** RVG/siNC (50 μg) and RVG/siPdcd4 (50 μg) were injected into mice through the tail vein, the changes of Pdcd4 mRNA level and BDNF expression in HIP and PFC were detected by RT-PCR and western blot 48 h later (*n* = 3 per group). **K**, **L** Under normal conditions RVG/siNC (50 μg) and RVG/siPdcd4 (50 μg) were injected into mice through the tail vein, and the control group was injected with equal volume of glucose solution. BDNF changes in HIP and PFC were measured by ELISA after injection for 48 h (*n* = 4 per group). Values are shown as mean ± SD, Student’s *t* test *p < 0.05. All the experiments were repeated at least three times independently.

Next, to prove the ability of RVG-9dR peptide on blood–brain barrier (BBB) traversing, Cy5 labeled-siPdcd4 was modified by RVG-9dR peptide (RVG/Cy5-siPdcd4) to visually track the movement of RVG/siRNA, and then the mixture was injected into mice through tail vein. Cy5 fluorescence signal was captured by imaging system to trace the distribution of Cy5-siPdcd4 in vivo. The results showed that only a small part of fluorescence was detected in brain at 6 h after intravenous administration (Fig. 4a). At 24 h after administration, Cy5 fluorescence was mainly concentrated in the brain, and the fluorescence intensity of the brain at 48 h is significantly weaker than 24 h. Eventually, there is no obvious fluorescence at 72 h after administration (Fig. 4b). Therefore, mice which were injected RVG-siRNA for 48 h was adopted for the next experiments. Then, the optimal concentration of siPdcd4 was tested, and the results indicated that 50 µg siPdcd4 has the similar effect as 75 µg in promoting BDNF (Fig. 4c, d). It means that 50 µg siPdcd4 injection was efficient and economical way to apply for silencing Pdcd4 in vivo. To detect the effect of RVG/siPdcd4 on the expression of BDNF, RVG/siNC, RVG/siPdcd4, or equal volume of 5% glucose solution was injected into mice, then Pdcd4 and BDNF levels in hippocampus and prefrontal cortex were detected by RT-PCR, western blot and ELISA after 48 h injection. The results showed that RVG/siPdcd4 significantly promoted BDNF expression while RVG/siNC did not affect BDNF expression in hippocampus and prefrontal cortex (Fig. 4e–l).

### RVG/siPdcd4 promotes BDNF expression and represses inflammation response during CRS

**Fig. 5:**
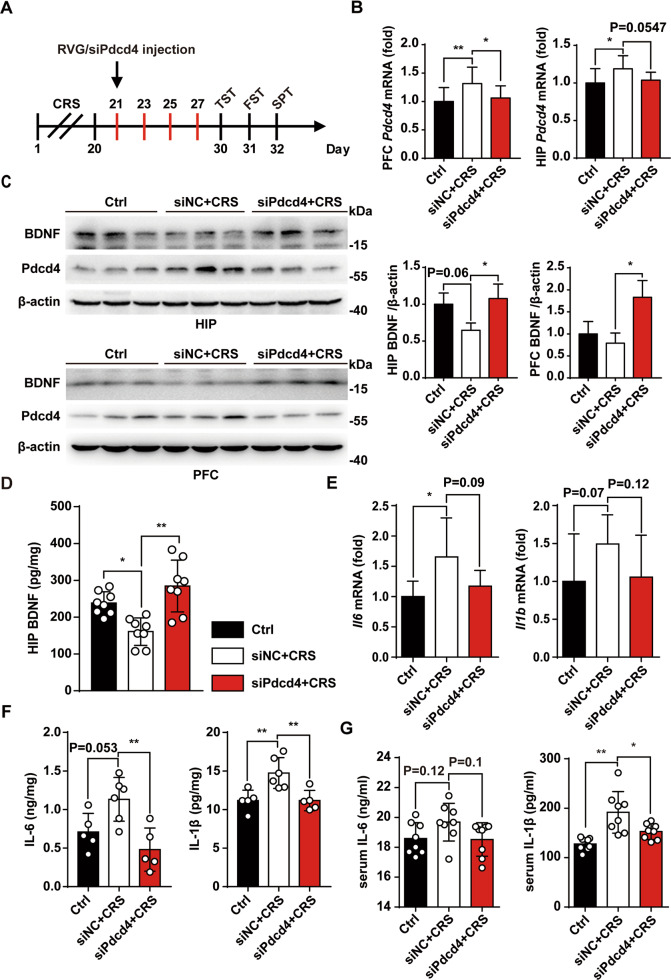
RVG/siPdcd4 promotes BDNF expression and represses inflammation response during CRS. **A** Mice depression model was induced by chronic restraint stress (CRS). RVG/siPdcd4 (50 μg, 2 mg/kg) was injected every other day from the 21st day. After the intervention of RVG/siPdcd4, mice depression behaviors were evaluated. **B** Fresh brain tissues were separated and total RNA were extracted using TRIzol reagent, and the changes of Pdcd4 mRNA level in HIP and PFC were detected by RT-PCR (*n* = 4 per group). **C** The protein changes of BDNF in the HIP and PFC were measured by western blot (*n* = 3 per group). **D** ELISA was used to analysis the changes of BDNF in HIP (*n* = 8 per group). **E** The changes of pro-inflammatory cyokines IL-6 and IL-1β mRNA level in HIP were detected by RT-PCR (IL-6 *n* = 7 per group, IL-1β *n* = 5 per group). **F** ELISA were used to analysis the changes of IL-6 and IL-1β expression in HIP (Ctrl and siPdcd4+CRS *n* = 5 per group, siNC+CRS *n* = 6). **G** ELISA were used to analysis the changes of IL-6 and IL-1β expression in serum (*n* = 8 per group). Values are indicated as mean ± SD, one-way ANOVA and multiple comparison test, *p < 0.05 **p < 0.01. All the experiments were repeated at least three times independently.

Chronic restraint stress (CRS) is an established model for mice depression. Our previous work has shown that CRS could up-regulated the expression of Pdcd4 that contribute to synaptic plasticity impairment and depression-like behavior by inhibiting the expression of BDNF [15]. To address the potential of RVG/siPdcd4 for depression therapy, RVG/siPdcd4 (2 mg/kg) was injected into depressive mice via tail vein, every 2 days for total of four times (Fig. 5a). Firstly, we analyzed the silencing effect of RVG/siPdcd4 treatment on Pdcd4 expression in depression related regions by RT-PCR. The results showed that RVG/siPdcd4 successfully knocked-down the expression of Pdcd4 in hippocampus and prefrontal cortex (Fig. 5b), whereas that resisted the reduction of BDNF induced by CRS in hippocampus and prefrontal cortex (Fig. 5c). The results of ELISA also showed that the expression of BDNF was significantly increased in hippocampus of RVG/siPdcd4 group (Fig. 5d). Furthermore, in order to evaluate the changes of inflammatory level in depressive mice after RVG/siPdcd4 treatment, mRNA and protein level of IL-6 and IL-1β in hippocampus were detected. The results showed that CRS induced the up-regulation of IL-6 and IL-1β expression, while RVG/siPdcd4 intervention suppressed the expression of pro-inflammatory cytokines IL-6 and IL-1β (Fig. 5e, f). The results of ELISA in serum of depressive mice showed that IL-1β level was significantly decreased after RVG/siPdcd4 treatment (Fig. 5g). These results suggest that RVG/siPdcd4 could reverse the down-regulation of BDNF expression induced by CRS and inhibit the expression of pro-inflammatory cytokines.

### RVG/siPdcd4 sustains synaptic plasticity and protects mice from CRS-induced depressive behavior

**Fig. 6:**
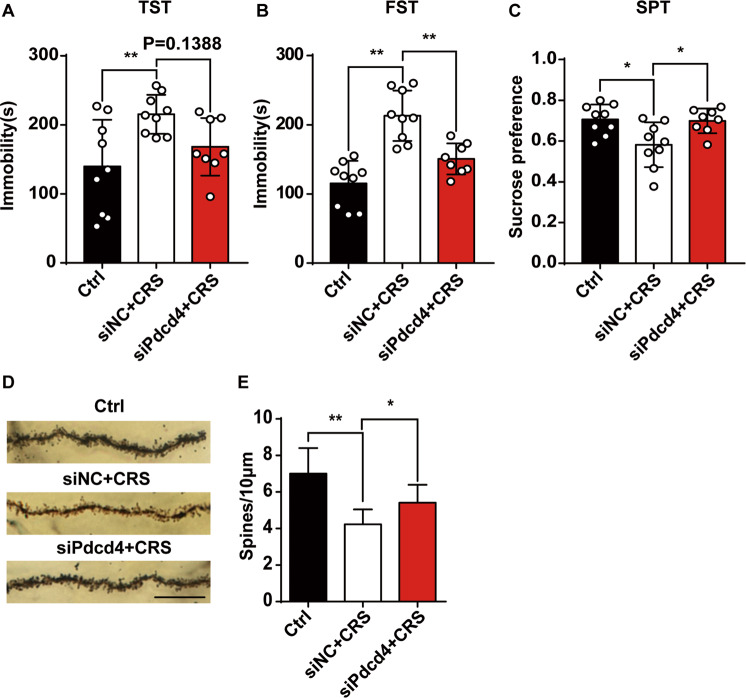
RVG/siPdcd4 sustains synaptic plasticity and protects mice from CRS-induced depressive behavior. **A** Immobility time in tail suspension test (TST) (Ctrl and siNC+CRS *n* = 9 per group, siPdcd4+CRS *n* = 8). **B** Immobility time in forced swimming test (FST) (Ctrl and siNC+CRS *n* = 9 per group, siPdcd4+CRS *n* = 8). **C** Sucrose consumption in Sucrose preference test (SPT) (Ctrl and siNC+CRS *n* = 9 per group, siPdcd4+CRS *n* = 8) were counted under normal, CRS and RVG/siPdcd4 treatment condition. **D** Representative photomicrographs of dendritic spines from DG granular cells, Scale bar, 20 μm. **E** Spine density in dendrites of DG granular cells, (Ctrl *n* = 8, siNC+CRS and siPdcd4+CRS *n* = 10 per group). Values are indicated as mean ± SD one-way ANOVA and multiple comparison test, *P ≤ 0.05 **P ≤ 0.01. All the experiments were repeated at least three times independently.

In order to evaluate the antidepressant effect of RVG/siPdcd4 treatment, behavioral tests were performed on depressive mice. The results showed that compared with CRS group the immobility time of RVG/siPdcd4 group in TST and FST were significantly reduced (Fig. 6a, b), and sucrose consumption index showed that RVG/siPdcd4 treatment rescued CRS-induced the decreased anhedonia of mice (Fig. 6c). These data demonstrated that RVG/siPdcd4 treatment could protect mice from CRS-induced depressive behavior. The synaptic plasticity impairment is an crucial pathological indicator of depression [28]. The dysfunction and reduction of BDNF has been correlated with neuronal atrophy and synaptic damage [29]. Golgi staining results showed that RVG/siPdcd4 administration obviously prevented neuron spines reduction caused by CRS (Fig. 6d, e). Collectively, our observations indicate that RVG/siPdcd4 effectively rescues CRS-induced neuronal atrophy and depression behavior via promoting BDNF expression.

## Discussion

In this study, we have demonstrated the potential of RVG/siRNA therapy to induce the knockdown of Pdcd4 in the CNS. More specifically, this therapeutic research has been proved to prevent synaptic lesion and BDNF expression abnormalities in CRS-induced depression model.

RVG peptide-guided siRNA system is firstly applied to depression treatment. RVG peptide is a rabies virus-derived 29 amino-acid residue fragment, and it has drawn much attention on the drug-modification research owing to its BBB penetrability [21]. Because the neuron-specific targeting advantage of RVG, systemic administration of the RVG/siRNA realizes brain local distribution of the therapeutic genes of interest. Although naked siRNA is rapidly destroyed by serum nucleases, we have confirmed that RVG/siRNA complex improves the stability of siRNA in vivo. In addition, our strategy enhances the bioavailability of siRNA since it is modified. Moreover, previous study has verified that the peptide also did not induce peptide-antibody responses and thus this method could be wildly used for neurological and psychiatric disorders treatment [21]. Therefore, RVG/siRNA system has designed for Alzheimer’s disease (AD) and Parkinson’s disease (PD) treatment. The dysfunction of BACE1 or the accumulation of α-Synuclein are the pathological characteristic of patients with AD or PD, and *siBACE1* or *sisynuclein* are developed to help these proteins degradation [23, 24]. However, due to the lack of specific gene therapy targets for depression, RVG/siRNA system has not been used for depression. Our previous report points that the overexpression of Pdcd4 leads to depressive behavior, and lentivirus packaged siPdcd4 is injected into hippocampus, that provids opportunity for depression therapy. In this article, we take advanced gene modification theory into rescuing depressive behavior, and found RVG/siPdcd4 system is a practical way to realize it.

siPdcd4, as a new type of antidepressant, could be applied to depression molecular therapy. Based on the monoamine hypothesis of depression, we have developed conventional antidepressants. However, with these drugs wildly used, the drawbacks are becoming more and more manifest. (1) the delayed onset is associated with an increased risk of suicidal behavior in the first month of antidepressant treatment. (2) approximately one-third depressed patients fail to respond to multiple antidepressant treatments and are considered treatment-resistant depression [30]. Ketamine, an *N*-methyl-d-aspartate receptor (NMDAR) antagonist, as a rapid-acting antidepressant, has been approved for depression treatment by the FDA in 2019 [31]. With different mechanisms from conventional antidepressants, the advantages of ketamine are effective and rapid-acting agent. From the study of ketamine anti-depressive effect, we learned the critical role of neurotrophic factors, such as BDNF [32]. Based on the molecular mechanism underlying the action of rapid-antidepressant, our research focuses on the regulation of BDNF expression in depression, and firstly discover a siRNA drug for depression treatment. siRNA drugs rapidly up-regulate the expression of BDNF by silencing specific target molecule, which is different from typical monoaminergic antidepressants. In addition, ketamine is reported to promote BDNF translation via inhibiting of elongation factor 2 (EF2) in the hippocampus [8, 33]. Our previous study also emphasized the critical translational mechanism of BDNF in depression, and found Pdcd4 is the key regulator in that. With the injection of Pdcd4 siRNA, we clearly found the increasing expression of BDNF in hippocampus, and the mice have anti-depressive behaviors [15]. Therefore, Pdcd4 is a typical molecule therapeutic target for depression and silencing Pdcd4 could specifically elevated BDNF expression at translational level. Besides that, a subset of depressed individuals has an inflammatory component that might be driving their disease process. Ketamine administration decreased the levels of IL-6, TNF-α, and IL-1β and suppressed the activation of NF-κB in depressed mice, suggesting pro-inflammatory inhibition plays an important role in depression treatment [34, 35]. In addition, in rodents’ model of depression, BDNF effectively prohibited cytokines by down-regulating NF-κB signaling. In this paper, the lower level of IL-6 and IL-1β in siPdcd4 injection group may be caused by BDNF expression or the directly effect of siPdcd4 on inflammation [36, 37]. In summary, siPdcd4, has multiple antidepressant effect and less side effect than any depression treatment drugs.

## Conclusion

In summary, we have shown that RVG-9dR provides a method for siPdcd4 delivery to brain, and it fully has antidepressant effect via promoting BDNF expression. These finding have solved the problem of neuro-targeted drug delivery in vivo, significantly accelerating the prospect of siRNA-based therapeutic for depression.

## Supplementary information

Supplementary information

## Data Availability

All data needed to support the conclusions of this article are present in the article.
