# Supplementary material for: The brain targeted delivery of programmed cell death 4 specific siRNA protects mice from CRS-induced depressive behavior
Source: Cell Death Dis. 2021 Nov 12;12(11):1077. doi: 10.1038/s41419-021-04361-9 (PMC8590023; doi:10.1038/s41419-021-04361-9)
Supplement: Supplementary file 1 — Supplementary information [file 41419_2021_4361_MOESM1_ESM.docx]

**Supplementary materials and methods**

**Tail suspension test (TST)**

The head of mice was suspended downward, and the tail was connected with the suspension frame by tape. The distance between the nose tip of mice and the equipment floor was 20-25 centimeter after suspension. The immobility time of mice was counted within 6 min, and the whole experiment process is videotaped. Result statistics and analysis are determined by Smart 3.0 video-tracking system (Harvard Apparatus, Massachusetts, USA).

**Forced swimming test (FST)**

Mouse was individually put into a transparent beaker (30 × 18 cm diameter) containing 16cm water (22 ± 0.5 °C). The time of mouse struggling to swimming and floating in the water within 6 min was counted, and the whole experiment process is videotaped. Result statistics and analysis are determined by Smart 3.0 video-tracking system (Harvard Apparatus, Massachusetts, USA).

**Sucrose preference test (SPT)**

The mouse was raised in a single cage. During the training period (two days), each mouse has two identical bottles containing 1% sucrose solution, and it is free to access water for 2 h (9:00-11:00.am). To avoid bottle side preference, the two bottles were switched in second day. In the formal experiment (two days), the mice were allowed free access to 1% sucrose solution and DDW from two different bottles for 2 h (9:00-11:00.am) on the third day, to avoid bottle side preference, the two bottles were switched in fourth day. Finally, the DDW and 1% sucrose solution consumption were measured and indicated by the weight loss of the bottles. Sucrose preference was calculated as follows: Sucrose preference (%) = sucrose consumption/ total consumption × 100%.

**Peptide and siRNA modification**

RVG-9dR (YTIWMPENPRPGTPCDIFTNSRGKRASNGGGGRRRRRRRRR) peptide was synthesized and purified by high-performance liquid chromatography (HPLC) at the GL Biochem ltd (Shanghai, China), and the purity of the RVG-9dR peptide used in the experiment is above 95%. The carboxy-terminal of RVG-9dR was nine D-arginine residues, and it was conjugated with Biotin at the amino terminus. Murine siRNAs used in the studies as follows: siNC (5’-UUCUCCGAACGUGUCACGUTT-3’) and siPdcd4 (5’-GAGGCUAUGAGAGAAUUUATT-3’) were synthesized in Gene Pharma (Shanghai, China). For the preparation of siPdcd4 with RVG-9dR peptide complex (RVG/siPdcd4), siPdcd4 and RVG-9dR peptide were mixed in a molar ratio of 1:10 in the opti-MEM medium (*in vitro*) or in 5% glucose solution (*in vivo*) for 20 min at room temperature. siPdcd4 was labelled with Cy5 at the 3’-end of the sense strand *in vivo* imaging experiments.

**In vivo imaging assay**

The mice were anesthetized 30 min before the *in vivo* observation. In order to eliminate the blocking effect of black hair on red fluorescence, the hairs of mice were removed with shaver. Meanwhile, mice front and rear limbs wiped with 75% ethanol to prevent the spontaneous fluorescence.50μg (RVG/Cy5-siPdcd4) was dissolved in 200μl 5% glucose solution and injected into mice through the tail vein. After 6h, 24h, 48h and 72h intravenous administration, the pictures of the distribution of red fluorescence in mice was captured by IVIS Lumina II *vivo* imaging system (PerkinElmer, MA, USA) at Translational Medicine Center Facility of Shandong University.

**Animal injection of RVG/siRNA complex**

RVG/siRNA intervention under normal condition. 50μg (2mg/kg) RVG/siNC and RVG/siPdcd4 were injected into mice through tail vein, mice in control group were injected with equal volume of 5% glucose solution.

RVG/siRNA intervention under chronic restraint stress condition. 50μg (2mg/kg) RVG/siPdcd4 dissolve in 200μl of 5% glucose was injected into mice through tail vein, every other day for 4 times. Behavioral tests and tissue analysis were performed after the RVG/siRNA intervention.

**Cell culture and siRNA transfection**

Human neuroblastoma cell line SH-SY5Y，mouse microglia cell line BV2 and mouse hippocampal neuron cell line HT-22 were purchased from Wuhan Typical Culture Preservation Center (Wuhan, Hubei, China). The cells were cultured in DMEM-F12(1:1) Medium (Corning, MA, USA) containing 10% fetal bovine serum (BI, Israel) in a standard incubator with an atmosphere of 5% CO_2_. Human cervical epithelial cancer cells (Hela) were cultured in Dulbecco’s Modified Eagle Medium (Corning, MA, USA) supplemented with10% fetal bovine serum (Corning, MA, USA). The primary hippocampal neurons were obtained from the hippocampus of fetal mice at the 16 days of pregnancy. The hippocampal tissue of fetal mice was digested into single cell suspension after 20 min of 0.25% trypsin treatment at 37°C, and the neuron cells were plated on the plates which were coated with 0.1 mg/ml poly-D-lysine (Sigma-Aldrich, MA, USA) in 1×10^6^ cells per well (6-well plant). Neurons were cultured in Neurobasal Medium supplemented with 2% B27 (Gibco, CA, USA) and L-glutamine (Thermo Scientific, CA, USA). The medium was changed into half liquid every other day and cultured for 7 days *in vitro*.

For the transfection, cells were plated in 6-well plates at 5 ×10^5^ cells per well and 100pM of siPdcd4 was transfected into cells with the help of lipofectamine 2000 (Invitrogen, CA, USA) when the density reached to 80% accordance with the manufacturer’s instructions.

**Western blot**

Cells and fresh brain tissue were washed with PBS to remove the impurities and placed on ice immediately. Cells and tissues in frozen RIPA lysis buffer (Beyotime, Beijing, China) containing cooktail proteinase inhibitor (Bimake, Shanghai, China) were fully lysed by tissue ultrasonic grinder (Servicebio, Wuhan, Hubei, China). The lysate were centrifuged at 13000 rpm for 20 min at 4 °C, and the supernatants were collected. Bicinchoninic acid (BCA) method was used to measure the concentration of protein using the BCA protein assay kit (Thermo Scientific, CA, USA). The proteins (30μg/lane) in each sample were separated on 15% sodium dodecylsulfate-polyacrylamide gels (SDS-PAGE) by electrophoresis and then the proteins were transferred to polyvinylidene difluoride (PVDF, Millipore, MA, USA) membranes using Semi-dry transfer method (15V, 1h). After blocking with 5% BSA for 1.5 h in room temperature, the membrane was incubated with primary antibodies overnight at 4 °C. The secondary HRP-conjugated antibodies were incubated for 1.5 h at room temperature with shaking. Bands were visualized using an enhanced chemiluminescence (ECL) (Millipore, MA, USA). The relative quantity of proteins was analyzed using Image J software (NIH, USA) and normalized to the control group.

Antibodies in this study:

| **ANTIBODYIES** | **SOURCE** | **IDENTIFIER** |
| --- | --- | --- |
| Anti-Pdcd4 | Cell Signaling Technology | #9535S |
| anti-β-actin | ZSGB-BIO | TA-09 |
| anti-BDNF | Proteintech | 28205-1-AP |
| HRP-conjugated anti-Rabbit | Jackson ImmunoResearch Laboratories | 323-005-024 |
| HRP-conjugated anti-mouse | Jackson ImmunoResearch Laboratories | 223-005-024 |

**Real time-PCR**

The total RNAs of cells were extracted according to the instruction of total RNA Extraction kit (Fastagen 2000, Shanghai, China). The total RNAs of fresh brain tissue were extracted using TRIzol Reagent (Invitrogen, CA, USA) according to the manufacturer’s instruction. Then the purified total RNAs (1000 ng) were reverse- transcribed to cDNA using the ReverTra Ace® qPCR RT Kit (TOYOBO, Tokyo, Japan). RT-PCR was performed on Bio-Rad iCycler (Bio-Rad, CA, USA). The Amplification conditions were as follows: 35 cycles of denaturation at 95 °C for 40 seconds, annealing at 55 °C for 30 seconds, and extension at 72 °C for 30 seconds. Relative target gene mRNA expression was normalized to β-actin mRNA and calculated using the△Ct method.

Primers used in this study:

| **Gene** | **Primer sequences** |
| --- | --- |
| Pdcd4 | Forward 5' AAACAACTCCGTGATCTTTGTCCA 3' |
|  | Reverse 5' TCAGGTTTAAGACGGCCTCCA 3' |
| IL-6 | Forward 5' CTGCAAGAGACTTCCATCCAG 3' |
|  | Reverse 5' AGTGGTATAGACAGGTCTGTTGG 3' |
| IL-1β | Forward 5' GCAACTGTTCCTGAACTCAACT 3' |
|  | Reverse 5' ATCTTTTGGGGTCCGTCAACT3' |
| β-actin | Forward 5' CAACTTGATGTATGAAGGCTTTGGT 3' |
|  | Reverse 5' ACTTTTATTGGTCTCAAGTCAGTGTACAG 3' |

**ELISA**

For the detection of IL-6, IL-1β and BDNF in cell culture supernatants or cell lysate, the cell fragments were removed by centrifugation at 13000rpm for 20 min at 4 °C. The concentration of IL-6, IL-1β and BDNF were quantified by specific Enzyme-Linked Immunosorbent Assay (ELISA) kits (Biolegend, CA, USA) according to the manufacturer’s instructions. The final concentration of cytokines and BDNF was measured using OD values.

**Golgi staining**

The mice were anesthetized by 5% chloral hydrate (7.5 ml/kg,i.p), and fresh brain tissue were obtained and washed with PBS. All the steps were followed on the manufacture instruction of TM FD Rapid GolgiStain Kit (FD NeuroTechnologies, MD, USA). Z-stack pictures (40×) were captured by Panoramic digital slice scanning microscope (VS120, OLYMPUS, Janpan). The number of nerve dendritic spines per 10 microns was counted.

**Statistical analysis**

The statistical analysis was performed using PrismGraphPad 7 Software. For comparisons of means between two groups two-tailed unpaired Students’ t test was performed. For comparisons of three groups One-way ANOVA were carried out by using Tukey’s post hoc test. The results are considered significant when the p value is <0.05*, <0.01**. All the data with normal distribution are presented as mean ± SD.
